# Molecular epidemiology of an extended multiple-species OXA-48 CPE outbreak in a hospital ward in Ireland, 2018–2019

**DOI:** 10.1017/ash.2021.206

**Published:** 2021-12-08

**Authors:** Carina Brehony, Lisa Domegan, Margaret Foley, Margaret Fitzpatrick, Jacqueline P. Cafferkey, Karina O’Connell, Binu Dinesh, Eleanor McNamara, Fionnuala Duffy, Fidelma Fitzpatrick, Karen Burns

**Affiliations:** 1 European Public Health Microbiology Training (EUPHEM), European Centre for Disease Prevention and Control, (ECDC), Stockholm, Sweden; 2 Public Health Laboratory, Cherry Orchard, Health Service Executive, Dublin, Ireland; 3 European Programme for Intervention Epidemiology Training (EPIET), European Centre for Disease Prevention and Control, (ECDC), Stockholm, Sweden; 4 Health Service Executive, Health Protection Surveillance Centre, Dublin, Ireland; 5 Department of Clinical Microbiology, Infection Prevention & Control, Beaumont Hospital, Dublin, Ireland; 6 Department of Clinical Microbiology, Royal College of Surgeons in Ireland, Dublin, Ireland

## Abstract

**Objectives:**

Molecular epidemiological description of an OXA-48 CPE outbreak affecting a tertiary-care hospital ward in Ireland over an extended period (2018–2019).

**Methods:**

Microbiological testing and whole-genome sequencing (WGS) were performed on all 56 positive OXA-48 outbreak case isolates.

**Results:**

In total, 7 different species were identified: *Enterobacter hormaechei* (n = 35, 62.5%), *Escherichia coli* (n = 12, 21.4%), *Klebsiella pneumoniae* (n = 5, 8.9%), *Klebsiella oxytoca* (n = 1, 1.8%), *Klebsiella michiganensis* (n = 1, 1.8%), *Citrobacter freundii* (n = 1, 1.8%), and *Serratia marcesens* (n = 1, 1.8%). *E. hormaechei* ST78 was the most common genotype (n = 14, 25%). Two major pOXA-48 plasmid types were identified throughout the outbreak, ‘types’ 1 and 2, and 5 major *E. hormaechei* clonal groupings were identified: ST78, ST108, ST1126, ST135, and ST66. Within each of the ST108, ST1126, ST135 and ST66 groups, the pOXA-48 harbored within each isolate were the same. Within ST78, 9 isolates contained the pOXA48 ‘type 2’ plasmid and 5 contained the ‘type 1’ plasmid. Environmental specimens were taken from different outbreak ward locations: handwash basins, sink and shower drains, and taps. Of 394 environmental specimens, OXA-48 CPE was isolated from 26 (6.6%).

**Conclusions:**

This prolonged outbreak of OXA-48 CPE was confined to one ward, but it exemplifies the complexity and difficulty in the control of these organisms. With multiple species and genotypes involved, they may be better described as ‘plasmid outbreaks.’ WGS provided insights into this diversity and potential transmission among cases, though its usefulness would be enhanced by analysis as close as possible to real time so that interventions can be implemented as soon as data are available.

Carbapenemase-producing Enterobacterales (CPE) infections can lead to treatment failure, extended hospital stays, increased healthcare costs, and increased mortality.^1^ They represent a major public health threat worldwide, and the World Health Organization has stated that research and development of antibiotics against them is of critical international importance.^2^ CPE infection has increased in prevalence globally since the early 2000s,^3–5^ when these organisms first emerged and they have been associated with nosocomial outbreaks in many countries including Ireland.^6,7^ As a result, CPE was declared a national public health emergency in Ireland in October 2017,^8^ and a concerted national effort to reduce the incidence of CPE demonstrated some early signs of success.^9^ OXA-48 CPEs have been particularly effective at spreading globally such that they are now the most common carbapenemase type in many countries, including Ireland.^9–11^

Here, we describe an outbreak of OXA-48 CPE affecting one ward in a tertiary-care hospital in Ireland over an extended period. The ward accommodated patients admitted under a variety of medical specialties and comprised a total of 35 beds, 5 of which were in single-bed rooms, whereas the remaining 30 beds were distributed among 4 six-bed rooms, 1 four-bed room, and 1 two-bed room. All patients admitted to the ward between July 2018 and December 2019 were included in this retrospective descriptive analysis. Outbreak cases were defined as patients admitted to the ward after July 1, 2018, who had a negative OXA-48 CPE admission screen (within 24 hours of admission) but subsequently had a positive microbiological specimen culture for OXA-48 CPE (n = 45). Here, we describe this lengthy CPE outbreak using microbiological and whole-genome sequencing (WGS) data.

## Methods

### Microbiological testing

All antimicrobial susceptibility testing was carried out according to the European Committee on Antimicrobial Susceptibility Testing (EUCAST) guidelines. Confirmation of CPE clinical and environmental isolates was conducted in the hospital clinical microbiology laboratory using: meropenem and ertapenem minimum inhibitory concentration results; CHROMID CARBA SMART screening plates (bioMérieux, Marcy-l'Étoile, France); immunochromatography using the RESIST-3 O.K.N. *K*-SeT flow assay (Coris BioConcept, Gembloux, Belgium) to detect OXA-48, KPC, and NDM carbapenemases; and PCR via the Xpert Carba-R assay (Cepheid, Sunnyvale, CA). Species identification was performed using matrix-assisted laser desorption/ionization time-of-flight mass spectroscopy (MALDI-TOF MS). Any isolates that were CPE negative on RESIST-3 O.K.N. *K*-SeT and PCR but were phenotypically nonsusceptible to carbapenems underwent carbapenem inactivation method (CIM) testing.^12^ Isolate species were identified using MALDI-TOF. CIM-positive isolates were referred to the National CPE Reference Laboratory Service (NCPERLS) for WGS and determination of other CPE types not tested for by the Xpert Carba-R platform.

### Environmental specimens

Although it was not undertaken during the early phase of the outbreak, environmental testing was conducted from November 2018 to May 2019 as part of outbreak investigation and ongoing monitoring. Environmental specimens for CPE detection were taken from a variety of locations within the ward. The focus of environmental sampling was high-touch surfaces, sinks, showers, and drains. Environmental sampling was not random and was not systematically conducted.

### WGS and bioinformatics analysis

All outbreak cases and 3 of the environmental isolates were sequenced (paired-end sequencing, read length 300 base pairs) using the Illumina MiSeq platform at NCPERLS. The resulting short reads were quality checked and assembled *de novo* using Spades within the BioNumerics (Applied Maths) genomics software platform. In the analysis, we also included 3 OXA-48 isolate genomes from the same hospital but not the same ward and with no identifiable epidemiological link to the outbreak ward. Assembled WGS data from NCPERLS were analyzed using BioNumerics to verify sequence quality, species, OXA-48 CPE gene presence, and genotype. We also described genetic relationships among isolates using a multilocus sequence type (MLST) gene-by-gene approach. Minimum-spanning trees of pOXA-48 locus (n = 71), ribosomal MLST (rMLST) locus (n = 53) and whole genome MLST (wgMLST) locus differences among isolates were constructed. Further genomic species identification was carried out using the rMLST.org website.^13^

### Control measures

Outbreak control interventions implemented included admission and weekly CPE screening of patients admitted to the outbreak ward, and isolation or cohorting of patients colonized with CPE, depending on availability of single rooms on the ward. Patient care encounters were undertaken using contact precautions, which included gloves and long-sleeved gowns for routine care. Proactive antimicrobial stewardship rounds were also undertaken. Enhanced environmental cleaning and disinfection was routinely implemented, along with hydrogen peroxide vapor (HPV) treatment upon patient discharge and regular HPV treatment of patient bathrooms and the sluice room. The ward was closed on several occasions, then decanted, with enhanced environmental cleaning and HPV treatment of the entire ward prior to reopening. Additionally, refurbishment works were undertaken across the ward, including patient bathrooms. In keeping with national guidance, staff screening for CPE carriage was not undertaken.

## Results

### Species identification

From 45 cases, a total of 59 new CPE isolates were obtained, with specimen dates between July 2018 and August 2019: 57 were from rectal swabs obtained during active surveillance cultures; 1 was obtained from a urine sample and 1 from a blood culture specimen. Moreover, 12 cases had >1 carbapenemase-producing species isolated (11 cases had 2 species and 1 case had 3 species) (Supplementary Table S1). MALDI-TOF was used to identify the species as follows: *Enterobacter cloacae* complex (n = 36, 61%), *Escherichia coli* (n = 14, 23.7%), *Klebsiella pneumoniae* (n = 5, 8.5%), *Klebsiella oxytoca* (n = 2, 3.4%), and *Citrobacter freundii* and *Serratia marcescens* (n = 1, 1.7%, respectively).

### Antimicrobial susceptibility testing

All isolates were nonsusceptible; they had intermediate or full resistance to amoxicillin, co-amoxiclav, and piperacillin/tazobactam (Supplementary Table S2). We also identified nonsusceptibility to gentamicin (n = 13, 22%), cotrimoxazole (n = 19, 32.2%), aztreonam (n = 23, 39%); and fosfomycin (n = 4, 6.8%). In total, 40 isolates (67.8%) were classified as extended-spectrum β-lactamase (ESBL) producers because they were nonsusceptible to 1 of the third- or fourth-generation cephalosporins tested for - cefotaxime, ceftazidime, or cefepime. In total, 54 isolates (91.5%) were nonsusceptible to ertapenem and 19 (32.2%) were nonsusceptible to meropenem. All isolates were susceptible to amikacin, ceftazidime/avibactam, and colistin.

### Genomic analysis

All isolates contained the *bla*_OXA-48_ carbapenemase-producing gene. Genomic species identification largely concurred with MALDI-TOF results (n = 56). However, WGS provided further definition for isolates identified as *Enterobacter cloacae* complex by MALDI-TOF (n = 35), identifying them all as *Enterobacter hormaechei*. One isolate identified as *K. oxytoca* was identified by WGS as *Klebsiella michiganensis*.

The 12 *E. coli* isolate genomes comprised 12 different sequence types (STs), one of which was a member of the globally spread multidrug-resistant clone, the ST131 complex. *E. hormaechei* isolates comprised 5 STs, of which ST78 was the most common (n = 14) and accounted for 40% of these isolates. Most ST78 isolates also appeared at a later point in the outbreak with 85.7% of this ST appearing from February 2019 onward (Table S3). *E. hormaechei* ST108 was the second most common (n = 7; 20%), but 4 of these isolates were sampled from 4 patients from a ward screen from the same day. All 3 environmental isolates referred for WGS were *K. michiganensis* ST143.

### OXA-48 outbreak strains species genotype diversity

**Fig. 1.**
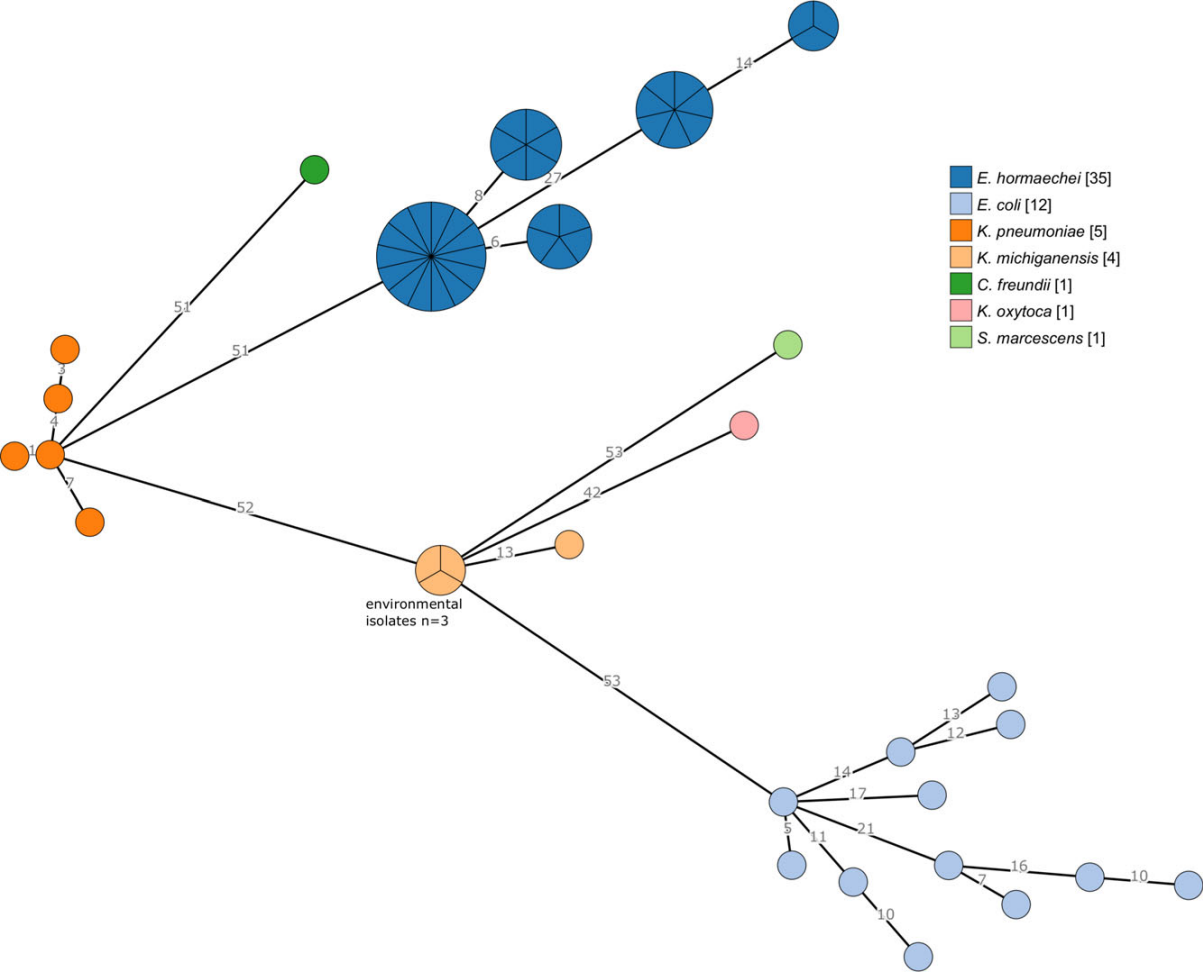
Minimum-spanning tree of ribosomal MLST locus (n = 53) allele differences among 56 outbreak cases and 3 environmental isolates. Each circle (node) contains isolates that are indistinguishable at all loci. Nodes are colored by species, as determined by WGS. Numbers on lines (edges) connecting nodes indicate the number of allele differences between connected nodes. Nodes are divided in pie-chart form for individual isolates.

We identified a diverse set of genotypes amongst the species, but *E. hormaechei* was less diverse with fewer ribosomal sequence types (rSTs), even though there were more isolates of this species (Fig. 1). All ST78 isolates shared the same rST56604 (n = 14) and all ST108 isolates shared the same rST63173 (n = 7). Indeed, isolates within each 7-locus ST also shared rSTs. Each of the *E. coli* isolates had a different rST. The 3 environmental *K. michiganensis* isolates shared the same rST but differed by 13 of 53 loci with the *K. michiganensis* case isolate.

### OXA-48 outbreak strain plasmid analysis

**Fig. 2.**
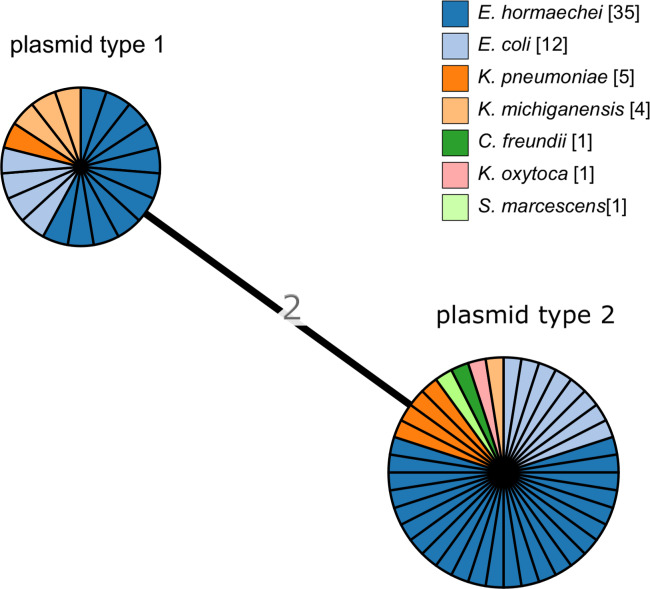
Minimum-spanning tree of pOXA-48 plasmid MLST locus (n = 71) allele differences among 56 outbreak cases and 3 environmental isolates. Each circle (node) contains isolates that are indistinguishable at all loci. Nodes are colored by species, as determined by WGS. Numbers on lines (edges) connecting nodes indicate the number of allele differences between connected nodes. Nodes are divided in pie-chart form for individual isolates.

**Fig. 3.**
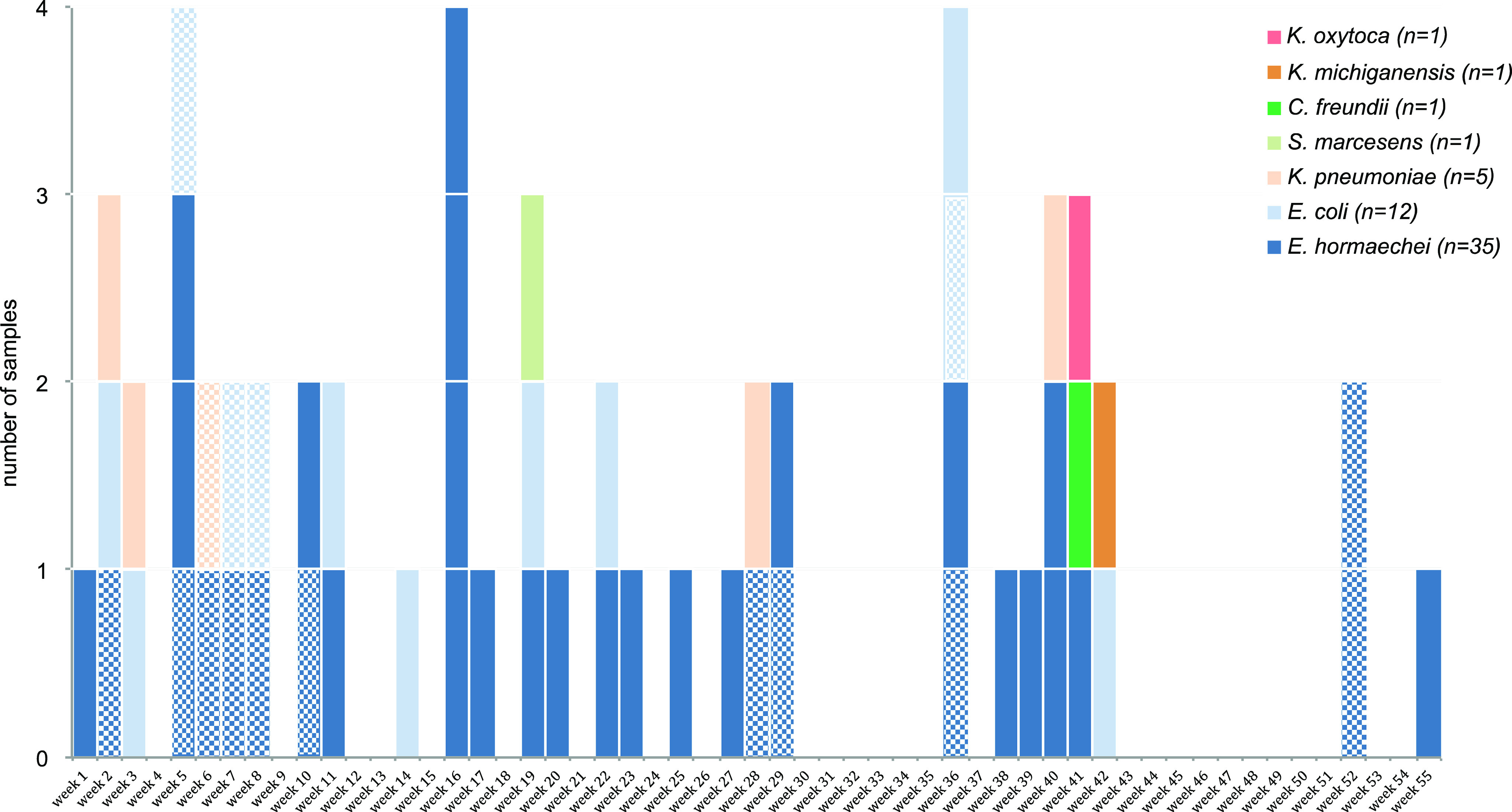
Species by week of outbreak that clinical specimen was taken (the week beginning July 29, 2018, to the week beginning August 11, 2019). Plasmid type 1 is indicated by checkered bars. All others plasmid are type 2.

We identified 2 major pOXA-48 plasmid types among the case isolates (Fig. 2), with 2 differences of the 71 pOXA-48 plasmid loci. There were 16 isolates (328.6%) with indistinguishable type 1 plasmids, and 40 isolates (71.4%) had indistinguishable type 2 plasmids. We detected no association with species; at least 4 of the species were represented by each plasmid type. The 2 plasmid types were contained in isolates from cases that spanned the whole outbreak period (Fig. 3). The environmental isolates (n = 3) all shared the same plasmid type, but the case isolate of the same species (ie, *K. michiganensis*) was the other plasmid type. Of the 12 cases with >1 species isolate, each pair of species, or 3 species in 1 patient, shared the same plasmid type. For example, in the patient from which *E. cloacae*, *E. coli*, and *S. marcescens* were isolated, all isolates harbored the type 2 plasmid. For 10 of 12 cases, the multiple species were isolated from specimens taken on the same date. For one case the second species was isolated a week later, and for another case the second species was isolated a month later.

### Outbreak Enterobacter cloacae complex and OXA-48 plasmid type

**Fig. 4.**
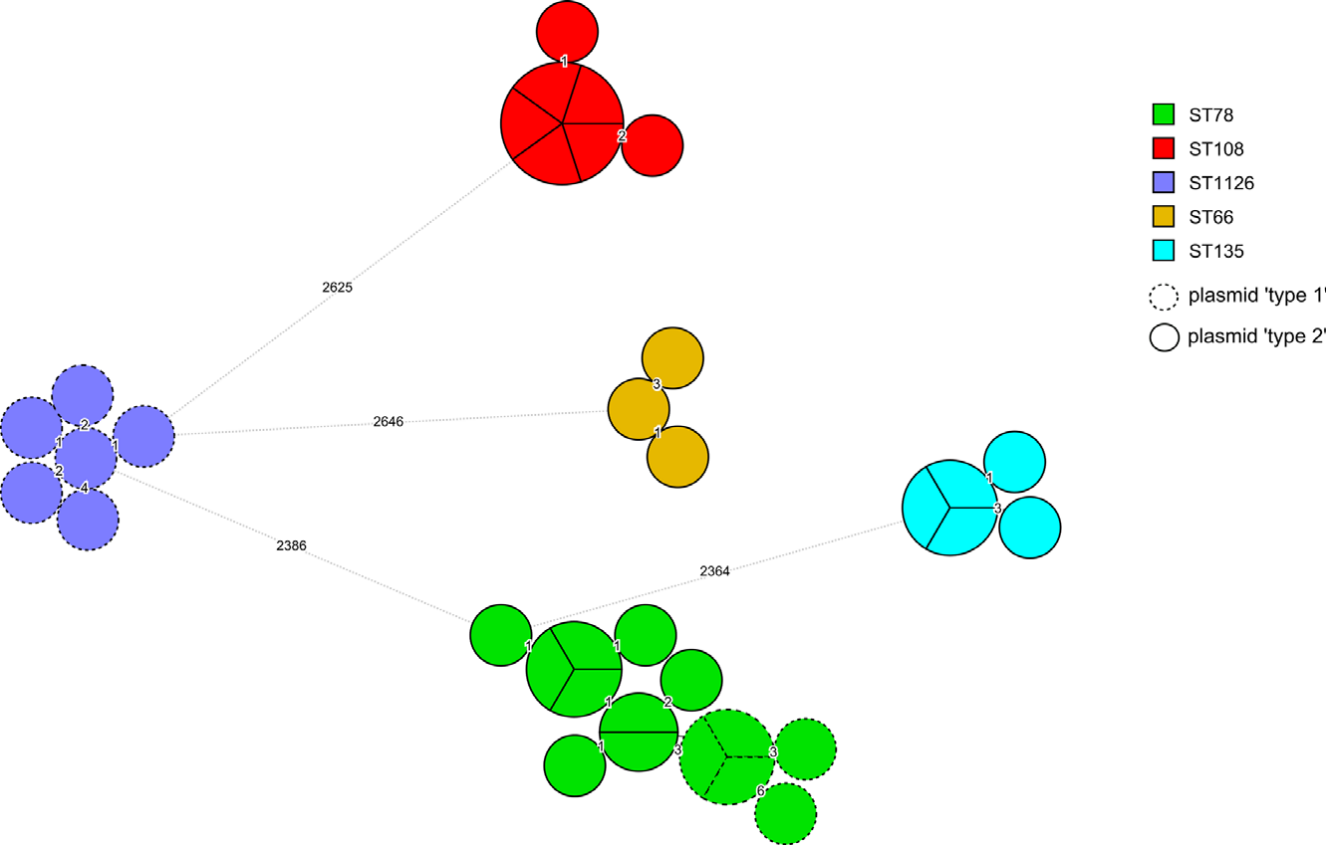
Minimum-spanning tree of *Enterobacter cloacae* complex wgMLST locus (n = 15,605) allele differences among 35 outbreak isolates. Each circle (node) contains isolates that are indistinguishable at all loci. Nodes are colored by 7 locus multilocus sequence types. Numbers on lines (edges) connecting nodes indicate the number of allele differences between connected nodes. Nodes are divided in pie-chart form for individual isolates.

We detected 5 major clonal groupings of *E. hormaechei:* ST78 (n = 14), ST108 (n = 7), ST1126 (n = 5), ST135 (n = 3), and ST66 (n = 3) (Fig. 4). Moreover, <7 locus differences among 15,605 wgMLST loci were identified within each grouping, suggesting a high degree of relatedness. Within each of the ST108, ST1126, ST135, and ST66 groups, the pOXA-48 harbored within each isolate was also the same type. Within the ST78 grouping, 9 isolates contained the type 2 plasmid and 5 contained the type 1 plasmid. Also, 3 *E. hormaechei* OXA-48 case isolates from patients accommodated elsewhere the hospital, with no known link to the outbreak ward, were clustered within the outbreak isolates and therefore were considered highly related. Two ST66 isolates from October and November 2018 were 1–2 wgMLST locus differences from other outbreak isolates. One ST78 isolate from October 2018 was 4 locus alleles different from the nearest case isolates. The more diverse ST78 was the longest persisting *E. hormaechei* genotype (Table S3). Except for 1 ST135 case in April 2019, none of the other STs (ST1126, ST108, or ST66) were associated with a case after February 2019.

### Environmental sampling

From November 2018 to May 2019, 394 environmental samples from the outbreak ward were tested (Supplementary Fig. S1). The earliest OXA-48–positive environmental sample was an OXA-48 *K. oxytoca* taken from the handwash basin tap in section F in January 2019. OXA-48 CPE was detected from 26 environmental samples (6.6%). The most common species detected were *E. cloacae* complex and *C. freundii*, each detected on 8 separate occasions. The last 2 positive environmental samples, on 2 separate dates in April 2019, were both *E. cloacae* complex. All positive samples were taken from handwash basins, sink drains, shower drains, or taps from various rooms or sections of the ward (Fig. S2). Of the locations in the ward with the highest number of positive environmental samples, section E was notable, with the most detections overall and detections on 9 separate occasions before and after the major decant and refurbishment in February 2019. Sink drains seemed to be particular hot spots, with OXA-48–producing isolates cultured from samples taken from sinks in several single-patient rooms as well as the treatment room (Supplementary Table S4).

## Discussion

This prolonged outbreak of OXA-48 CPE on a tertiary-care hospital ward exemplifies the complexity and difficulty in the control of these organisms. Nosocomial CPE outbreaks can involve single clones,^14,15^ but they can also involve multiple genotypes and species,^16^ making tracking of transmission very difficult. Clonal and nonclonal spread of OXA-48 in nosocomial outbreaks have been documented.^17^ Nosocomial CPE outbreaks may often also be protracted and last several years.^14,15,18^ Here, the first outbreak case presented on July 31, 2018, and the last was >1 year later on August 12, 2019. The outbreak involved 7 different *Enterobacterales* spp, and within each species no single clone predominated. However, the ST78 *E. hormaechei* genotype was the most prevalent overall and is a clone associated with nosocomial outbreaks and with other carbapenemase types.^15,19,20^ This ST was the most predominant in cases, despite major IPC measures, including a ward decant and refurbishment in February 2019.

Despite the complexity, genomics did provide some insight into the outbreak dynamics. Given that several patients harbored isolates that were indistinguishable by high-resolution genomics methods, there may have been short-chain transmission of the organism among patients or it may have been acquired from a common source, either environmental or another unknown and unsampled contact. For example, 5 cases shared the same ST108 *E. hormaechei* with the same pOXA-48 plasmid that were indistinguishable by wgMLST. Of these 5 cases, 4 were sampled on the same day and 1 isolate was from a sample taken ∼6 weeks later. A number of these cases were known to share a common contact and/or overlapped in their stay in a section of the ward. Also, 2 of these sections, D and F, had positive environmental screens (*K. oxytoca* and *E. cloacae* complex). The direction of potential transmission between patients or between patients and environment is difficult to ascertain, particularly in a retrospective analysis. Patient-to-patient transmission is known to occur, and these organisms, particularly *Klebsiella* and *Enterobacter* spp, have become adapted to the nosocomial environment. Thus, stringent adherence to all elements of standard and transmission-based precautions is required. Retrospective genomic analysis can highlight links between apparently sporadic CPE cases in the nosocomial environment.^21^

Two closely related yet distinct pOXA-48 plasmid types were identified in this outbreak. Both of these have been found across Ireland over the past several years,^22^ and one, called here ‘plasmid type 2,’ was associated with a prolonged outbreak in another large urban hospital in Ireland. Within-patient colonization of multiple OXA-48 species and interspecies plasmid transfer have been well documented.^23–25^ For all 12 cases with >1 species isolate, each pair of species, or 3 species in the case of one patient, shared the same plasmid type. In most of these cases, specimens were taken on the same date. This finding may indicate the sharing of plasmids between species within patients. Again, these results highlight the importance of infection prevention precautions to prevent opportunities for colonization. However, no means for decolonizing CPE carriers is available yet, so measures to reduce and prevent infection (eg, wound and invasive device management) are of critical importance.

From the relatively short period during which environmental sampling was carried out, 5 species were detected from various locations within the ward. Despite many outbreak control interventions and extensive refurbishment of the ward, including patient bathrooms, a number of positive environmental screens were obtained. With a total of 26 environmental OXA-48–positive samples detected on the outbreak ward during the outbreak period, the most likely source of exposure was environmental, along with person-to-person transmission between patients on the outbreak ward. An additional study combining social network analysis and genomics was undertaken to fully explore transmission patterns during this outbreak.

This study had several limitations. The environmental sampling on the outbreak ward was not initiated until later in the outbreak management and was not systematic. The study was limited to mainly one period in the spring of 2019, and only 3 of the OXA-48 isolates were referred for WGS. WGS data from all positive environmental isolates on the outbreak ward could have revealed further transmission patterns and links to or among outbreak cases and various locations on the outbreak ward.

The presence of closely related OXA-48 isolates from elsewhere in the hospital without identifiable or known epidemiological links to the outbreak ward raises the possibility that the outbreak may have spread beyond the outbreak ward. Alternatively, a small number of outbreak cases may have been exposed from a common source elsewhere in the hospital, or unrecognized person-to-person or equipment-to-person transmission within the hospital. This possibility also gives rise to the question of whether the case definition could have been expanded to other areas of the hospital. The further detailed investigation and follow-up of these nonoutbreak cases was outside the scope of this study.

Recommendations from this work, which may help to prevent or control future CPE outbreaks, include (1) implementation of regular systematic environmental sampling in the hospital in outbreak and nonoutbreak periods; (2) consideration of performing periodic point-prevalence surveys for CPE carriage across the hospital, with WGS and detailed analysis carried out on any positive sample isolates in real time, along with comparison between clinical and environmental sample results; and (3) continued compliance with national CPE clinical guidelines on patient screening for CPE carriage to ensure prompt detection of carriers. Where environmental sampling results yield CPE, interventions to eradicate CPE from those areas should be implemented, with replacement of sink and shower drain pipes that may aid prolonged survival of microorganisms if CPE is persistently cultured from those sites, despite interventions. Future research opportunities could include using metagenomics to index the diversity of microbes and antimicrobial and disinfectant resistance genes across a range of locations across the hospital and comparing these to patient microbiomes.^26^ Distinct ecological niches, many of which harbor stable populations over time, have been described in hospital environments such as high-touch surfaces and sink traps.^26,27^ Knowledge of the hospital microbiome can aid understanding of the biology of these organisms, potential nosocomial acquisition and transmission, as well as effectiveness of IPC measures. Although some of these recommendations may incur human and monetary resources that are already stretched, particularly in the wake of the COVID-19 pandemic, prevention of morbidity and mortality from infections caused by CPE would hopefully avoid a greater economic burden in the long term.

## Supplementary material

For supplementary material accompanying this paper visit https://doi.org/10.1017/ash.2021.206. click here to view supplementary material
