# Supplementary material for: Molecular epidemiology of an extended multiple-species OXA-48 CPE outbreak in a hospital ward in Ireland, 2018–2019
Source: Antimicrob Steward Healthc Epidemiol. 2021 Dec 8;1(1):e54. doi: 10.1017/ash.2021.206 (PMC9495434; doi:10.1017/ash.2021.206)
Supplement: Supplementary file 1 [file S2732494X21002060sup001.docx]

**Supplementary material**

**Table S1: OXA-48 outbreak cases with multiple species (n=12)**

| **species combination** | **no of cases** | |
| --- | --- | --- |
| *E. cloacae, E. coli* | 5 |  |
| *E. cloacae, K. pneumoniae* | 2 |  |
| *E. coli, K. pneumoniae* | 2 |  |
| *K. oxytoca, C. freundii* | 1 |  |
| *E. cloacae, K. oxytoca* | 1 |  |
| *E. cloacae, E. coli, S. marcescens* | 1 |  |

**Table S2: Number and percentage (in brackets) of non-susceptible *i.e.* intermediate or resistant case isolates (n=59) broken down by species. Antimicrobial susceptibility testing carried out as per EUCAST guidelines.**

| **species** | **amoxicillin** | **co-amoxiclav** | **pip/taz** | **ciprofloxaxin** | **gentamicin** | **co-trimoxazole** | **cefotaxime** | **ceftazidime** | **cefepime** | **fosfomycin** | **meropenem** | **ertapenem** | **aztreonam** |
| --- | --- | --- | --- | --- | --- | --- | --- | --- | --- | --- | --- | --- | --- |
| *E. cloacae* complex (n=36) | 36 (100) | 36 (100) | 36 (100) | 21 (58.3) | 13 (36.1) | 15 (41.6) | 36 (100) | 25 (69.4) | 26 (72.2) | 4 (11.1) | 12 (33.3) | 36 (100) | 22 (61.1) |
| *E. coli* (n=14) | 14 (100) | 14 (100) | 14 (100) | 1 (7.1) | 0 (0) | 3 (21.3) | 2 (14.2) | 1 (7.1) | 1 (7.1) | 0 (0) | 4 (28.6) | 10 (71.4) | 1 (7.1) |
| *K. pneumoniae* (n=5) | 5 (100) | 5 (100) | 5 (100) | 0 (0) | 0 (0) | 1 (20) | 0 (0) | 1 (20) | 0 (0) | 0 (0) | 1 (20) | 5 (100) | 0 (0) |
| *S. marcescens* (n=1) | 1 (100) | 1 (100) | 1 (100) | 0 (0) | 0 (0) | 0 (0) | 1 (100) | 0 (0) | 0 (0) | 0 (0) | 1 (100) | 1 (100) | 0 (0) |
| *C. freundii* (n=1) | 1 (100) | 1 (100) | 1 (100) | 0 (0) | 0 (0) | 0 (0) | 0 (0) | 0 (0) | 0 (0) | 0 (0) | 0 (0) | 1 (100) | 0 (0) |
| *K. oxytoca* (n=2) | 2 (100) | 2 (100) | 2 (100) | 0 (0) | 0 (0) | 0 (0) | 0 (0) | 0 (0) | 0 (0) | 0 (0) | 0 (0) | 2 (100) | 0 (0) |

Note: AST was carried also on isolates against amikacin, caz/avibactam and colistin but no isolates were non-susceptible and so are omitted from table to aid clarity of reading

**Table S3: Sequence Types (STs) of isolates from cases over outbreak time period by outbreak week. Week beginning 29/07/2018 (week 1) to week beginning 11/08/2019 (week 55)**

| Species | Sequence Type | wk 1 | wk 2 | wk 3 | wk 5 | wk 6 | wk 7 | wk 8 | wk 10 | wk 11 | wk 14 | wk 16 | wk 17 | wk 19 | wk 20 | wk 22 | wk 23 | wk 25 | wk 27 | wk 28 | wk 29 | wk 36 | wk 38 | wk 39 | wk 40 | wk 41 | wk 42 | wk 43 | wk 55 | Total |
| --- | --- | --- | --- | --- | --- | --- | --- | --- | --- | --- | --- | --- | --- | --- | --- | --- | --- | --- | --- | --- | --- | --- | --- | --- | --- | --- | --- | --- | --- | --- |
| *E. hormaechei* | ST66 | 1 |  |  | 1 |  |  |  |  |  |  |  |  |  |  |  |  |  | 1 |  |  |  |  |  |  |  |  |  |  | 3 |
|  | ST1126 |  | 1 |  | 1 | 1 | 1 | 1 | 1 |  |  |  |  |  |  |  |  |  |  |  | 1 |  |  |  |  |  |  |  |  | 7 |
|  | ST78 |  |  |  | 1 |  |  |  | 1 |  |  |  |  |  |  |  |  |  |  | 1 | 2 | 2 | 1 |  | 2 | 1 |  | 2 | 1 | 14 |
|  | ST108 |  |  |  |  |  |  |  |  |  |  | 4 |  |  |  |  | 1 | 1 |  |  |  |  |  |  |  |  |  |  |  | 6 |
|  | ST135 |  |  |  |  |  |  |  |  | 1 |  |  | 1 | 1 | 1 |  |  |  |  |  |  |  |  | 1 |  |  |  |  |  | 5 |
| *E. coli* | ST405 |  | 1 |  |  |  |  |  |  |  |  |  |  |  |  |  |  |  |  |  |  |  |  |  |  |  |  |  |  | 1 |
|  | ST131 |  |  | 1 |  |  |  |  |  |  |  |  |  |  |  |  |  |  |  |  |  |  |  |  |  |  |  |  |  | 1 |
|  | new |  |  |  | 1 |  |  |  |  |  |  |  |  | 1 |  |  |  |  |  |  |  |  |  |  |  |  |  |  |  | 2 |
|  | ST297 |  |  |  |  |  | 1 |  |  |  |  |  |  |  |  |  |  |  |  |  |  |  |  |  |  |  |  |  |  | 1 |
|  | ST59 |  |  |  |  |  |  | 1 |  |  |  |  |  |  |  |  |  |  |  |  |  |  |  |  |  |  |  |  |  | 1 |
|  | ST95 |  |  |  |  |  |  |  | 1 |  |  |  |  |  |  |  |  |  |  |  |  |  |  |  |  |  |  |  |  | 1 |
|  | ST538 |  |  |  |  |  |  |  |  | 1 |  |  |  |  |  |  |  |  |  |  |  |  |  |  |  |  |  |  |  | 1 |
|  | ST963 |  |  |  |  |  |  |  |  |  | 1 |  |  |  |  |  |  |  |  |  |  |  |  |  |  |  |  |  |  | 1 |
|  | ST537 |  |  |  |  |  |  |  |  |  |  |  |  |  |  | 1 |  |  |  |  |  |  |  |  |  |  |  |  |  | 1 |
|  | ST399 |  |  |  |  |  |  |  |  |  |  |  |  |  |  |  |  |  |  |  |  | 1 |  |  |  |  |  |  |  | 1 |
|  | ST718 |  |  |  |  |  |  |  |  |  |  |  |  |  |  |  |  |  |  |  |  |  |  |  |  |  | 1 |  |  | 1 |
|  | ST4274 |  | 1 |  |  |  |  |  |  |  |  |  |  |  |  |  |  |  |  |  |  |  |  |  |  |  |  |  |  | 1 |
| *K. pneumoniae* | ST252 |  |  | 1 |  | 1 |  |  |  |  |  |  |  |  |  |  |  |  |  |  |  |  |  |  |  |  |  |  |  | 2 |
|  | ST45 |  |  |  |  |  |  |  |  |  |  |  |  |  |  |  |  |  |  | 1 |  |  |  |  |  |  |  |  |  | 1 |
|  | ST412 |  |  |  |  |  |  |  |  |  |  |  |  |  |  |  |  |  |  |  |  |  |  |  | 1 |  |  |  |  | 1 |
| *K. michiganensis* | ST43 |  |  |  |  |  |  |  |  |  |  |  |  |  |  |  |  |  |  |  |  |  |  |  |  |  | 1 |  |  | 1 |
| *K. oxytoca* | ST37 |  |  |  |  |  |  |  |  |  |  |  |  |  |  |  |  |  |  |  |  |  |  |  |  | 1 |  |  |  | 1 |
| *S. marcesens* | na |  |  |  |  |  |  |  |  |  |  |  |  | 1 |  |  |  |  |  |  |  |  |  |  |  |  |  |  |  | 1 |
| *C. freundii* | ST62 |  |  |  |  |  |  |  |  |  |  |  |  |  |  |  |  |  |  |  |  |  |  |  |  | 1 |  |  |  | 1 |
|  | **Total** | **1** | **3** | **2** | **4** | **2** | **2** | **2** | **3** | **2** | **1** | **4** | **1** | **3** | **1** | **1** | **1** | **1** | **1** | **2** | **3** | **3** | **1** | **1** | **3** | **3** | **2** | **2** | **1** | **56** |

**Figure S1: Breakdown of environmental testing results and dates of environmental sampling on outbreak ward**

**Table S4: locations of positive environmental screens**

| **Sampling location** | **Count** |
| --- | --- |
| Patient Room 1: Handwash sink drain | 2 |
| Patient Room 2: Handwash sink drain | 4 |
| Patient Room 4: Handwash sink drain | 1 |
| Patient Room 5: Handwash sink drain | 1 |
| Section E: Handwash sink drain | 7 |
| Section F: Handwash sink tap | 1 |
| Treatment Room: Handwash sink drain | 2 |
| Treatment Room: Equipment side room sink drain | 1 |
| Section E: Bathroom sink tap | 1 |
| Patient Room 4: Bathroom sink tap | 1 |
| Patient Room 3: Shower drain | 1 |
| Section C: Shower drain | 3 |
| Section E: Shower drain | 1 |
| **Total** | **26** |

**
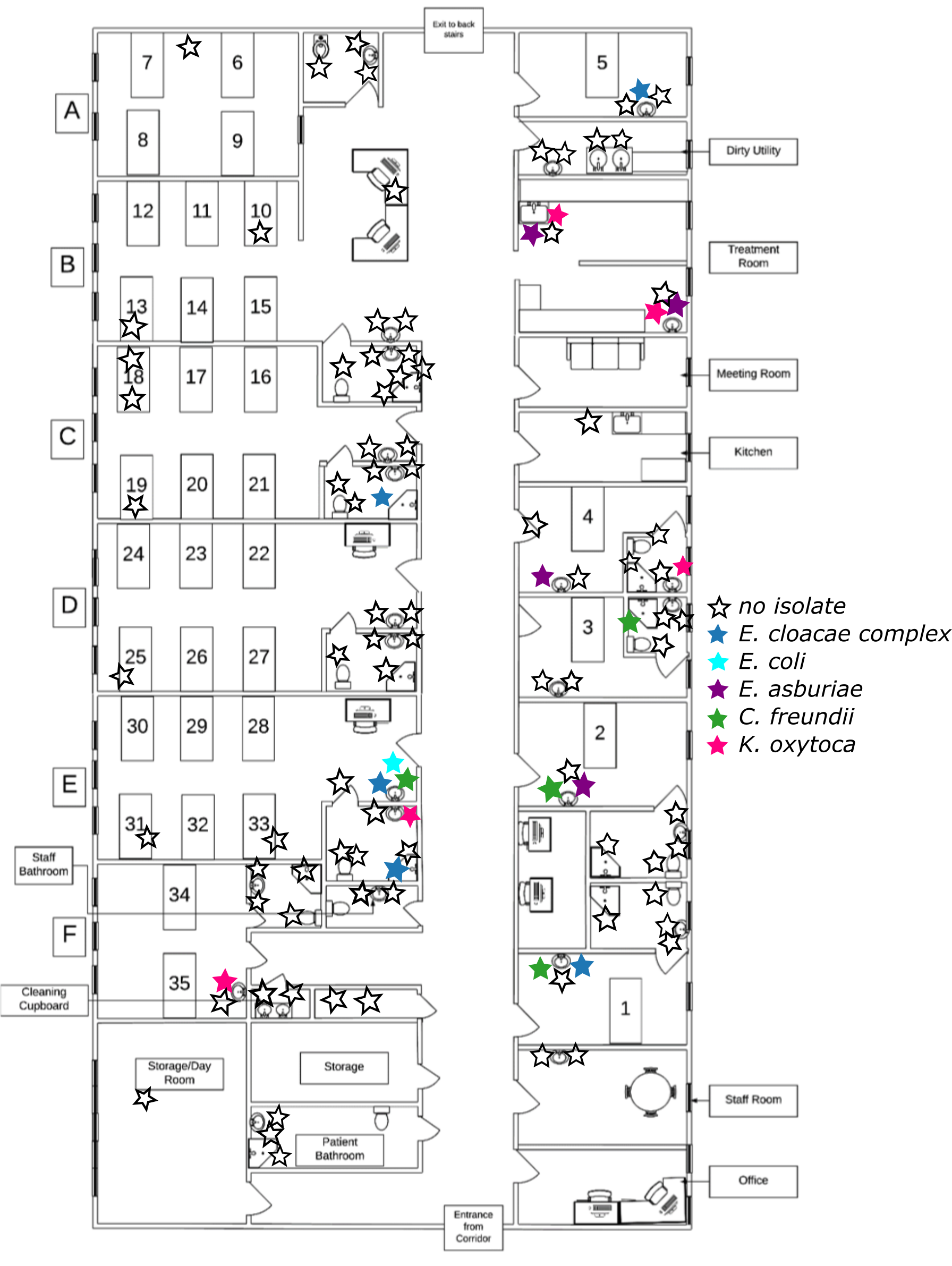
**

**Figure S2: plan of outbreak ward with locations of environment sampling. Note: Species identification by MALDI-ToF. Coloured stars indicate positive screen (see legend) and open stars indicate negative screen.**
